# Community medical centers struggle to produce well-calibrated clinical prediction models: Data augmentation can help

**DOI:** 10.1016/j.jbi.2026.105012

**Published:** 2026-03-11

**Authors:** Katherine E. Brown, Bradley A. Malin, Sharon E. Davis

**Affiliations:** Vanderbilt University Medical Center Department of Biomedical Informatics, 2525 West End Avenue Suite 1400, Nashville, 37203, TN, USA

**Keywords:** Machine learning, Synthetic data generation, Clinical prediction models, Community healthcare, 68T99, 68U99

## Abstract

**Objective:**

Machine learning models (ML) often require localization to perform optimally in local populations. We hypothesize that smaller community healthcare centers may not have the necessary patient volume to facilitate localization based on statistical guidelines. This work investigates the ability for community medical centers to localize ML and performs a simulation study to evaluate synthetic data generation (SDG) to augment local data for recalibration.

**Methods:**

We conducted an experiment using data from a real network of hospitals (two rural, one urban academic medical center) to predict 30-day unplanned hospital readmission and using data from a multi-site ICU dataset to simulate using synthetic data generation (SDG) in a network of hospitals of various sizes. We also performed a simulation study using data from a multi-site ICU dataset to evaluate the utility of SDG to augment local data volumes.

**Results:**

In the real-world evaluation, the urban medical center met the guidelines for the number of samples for recalibration (Required: 14,224, Available: 42,303) and had the best calibrated model using local data (α=0.1,β=1.05; best: α=0,β=1). For the smaller sites, neither site had the samples required for recalibration (Site 1: Required: 16461, Available: 3187; Site 2: Required: 15299, Available: 905). In the simulation study, deep learning-based SDG was most effective at improving calibration performance.

**Conclusions:**

Connections to large medical centers are not enough to promote accurate ML at all sites within a healthcare system. Data augmentation and SDG may provide the necessary data volumes to enable local recalibration at smaller facilities.

## Graphical Abstract

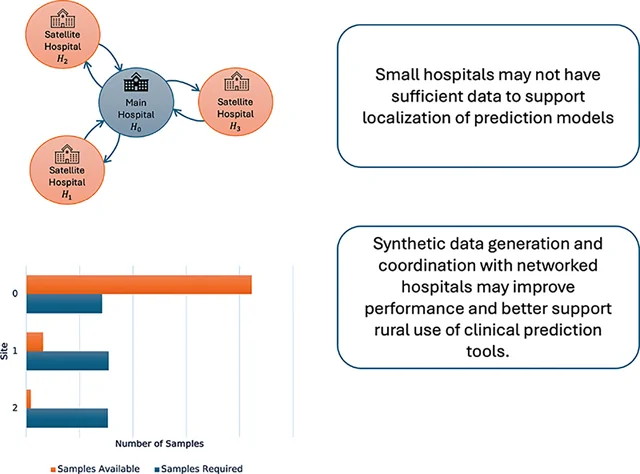

## 1. Introduction

Artificial intelligence (AI) tools, and particularly machine learning (ML) models, have increased in their adoption for a variety of clinical tasks, including predicting the likelihood of disease [1] or efficacy of medication [2] as well as assessing the likelihood of hospital discharge [3] or readmission [4]. Still, there are non-trivial challenges regarding the transportability of these models across sites [5–7]. As has been shown, a model trained at one site (e.g., hospital, floor, or ward) can deteriorate in overall reliability – measured as the calibration of model outputs to true likelihood of experiencing the outcome – when applied at other sites [8,9]. While generative AI models such as large language models may provide potential mitigation for performance drift, LLMs have been shown to struggle as clinical prediction models compared to traditional machine learning [10]. Moreover, machine learning may still be a more accessible and viable option for a variety of medical centers. Due to concerns regarding model transportability, guidelines recommend updating machine learning models using local site data [11–13]; however, it is possible that not all healthcare organizations, and especially those in smaller communities, have the patient volume necessary to facilitate a meaningful update.

Approximately 56,000,000 people in the United States (18% of the US population) reside in a rural area. With multiple financial and operational challenges facing this population, the promise of clinical prediction to advance care and improve efficiency is critical to enhancing lives and outcomes. While Austen and colleagues [14] proposed a framework to evaluate clinical prediction models across multiple geographic sites, there has been little investigation into the capabilities of smaller medical centers to update clinical prediction models for their local populations. As such, there is an urgent need to characterize the impact and applicability of model updating and recalibration guidelines on the smaller medical centers.

In this work, we illustrate existing capabilities of smaller medical centers to update clinical prediction models to ensure predictions are well-calibrated for their patients. Additionally, in a simulation study, we evaluate the possibility of synthetic data generation [15,16] to augment existing local data as an option to mitigate sample size concerns, extending previous studies utilizing data augmentation to mitigate equity concerns in AI models [17,18]. We specifically investigate the following research questions (RQs): **RQ 1:** How well can satellite healthcare facilities within a larger health system locally update clinical prediction models based on existing guidelines? **RQ 2:** How well can data augmentation —via synthetic data generation and oversampling existing data volumes —mitigate sample size concerns when updating clinical models at smaller sites compared to global data available across all sites?

**Table:** 

Statement of significance	
Problem	Clinical prediction models require recalibration for optimal performance in local populations; however, some community healthcare centers serve regions with sparser populations which could lead to an inability to properly recalibrate.
What is already known	Synthetic data generation (SDG) has been used to improve the algorithmic fairness of clinical prediction models. There are statistical guidelines that inform the number of samples needed to recalibrate a model for local populations. The ability for smaller medical centers serving sparse populations to meet these guidelines is underexplored.
What this paper adds	This paper presents an analysis of a real-world clinical prediction model currently in use at community medical centers. We perform a simulation study on a publicly available, multi-site clinical database to evaluate the potential of SDG approaches to meet guidelines for model recalibration.
Who would benefit from the new knowledge in this paper	Researchers working with synthetic data generation or optimization of clinical prediction models for sparse populations would benefit from the analysis of synthetic data generation for model recalibration.

## 2. Methods

This study was approved by the Vanderbilt University Medical Center Institutional Review Board (#240126).

Table 1 presents the formal mathematical framework used to model a multi-site network of hospitals consisting of a main hospital or academic medical center and n satellite hospitals serving a likely diverse population of local communities. A narrative description of this framework is provided in Appendix A.

Given this framework, we can formally describe our main hypotheses, with the corresponding research question in parentheses, as follows: **Hypothesis 1 (RQ 1):** For site $H_{i}$ where 1≤i≤n, the optimal calibrator for a machine learning algorithm will not be $l_{0}(\cdot )$ (e.g., the calibrator derived from the training data of the main hospital/site). **Hypothesis 2 (RQ 1):** Hospital $H_{i}$ will not have the required amount of data in $T_{{\mathrm{train}}_{i}}$ to produce the optimal calibrator $l_{i}(\cdot )$ for 1≤i≤n. **Hypothesis 3 (RQ 2):** Augmenting existing data in $T_{{\mathrm{train}}_{i}}$ will produce a more optimal $l_{i}(\cdot )$ compared to $l_{A}(\cdot )$ for 1≤i≤n

### 2.1. Experiment 1: Evaluation of real-world clinical prediction model at satellite hospitals.

For the remainder of this paper, we refer to this experiment as the Real-World Evaluation.

#### 2.1.1. Data and model

To characterize the ability of local sites to update and recalibrate clinical prediction models, we consider a model currently in production in a network of hospitals in the Middle Tennessee area (see Table 2). This network includes one urban academic medical center and two satellite hospitals in predominantly rural communities. Table 2 presents each site along with the population density of the county in which the site is located, area deprivation index [19] (ADI), and whether or not the site is eligible for Health Resources and Services Administration (HRSA) rural health funding [20]. The Area Deprivation Index (ADI) is a composite score of expected socioeconomic disadvantage for a geographic area. This measure considers information on income, education, employment, and housing quality collected through the U.S. Census and has been widely adopted in public health research [19]. Higher values of ADI indicate higher amounts of expected socioeconomic disadvantage which is associated with, for example, higher risk of hospital readmission [21]. HRSA directs programs to improve healthcare in the US to historically disadvantaged or underserved populations [22] including rural communities [23]. Table B.8 in Appendix B contextualizes the individual sites in this experiments based on the overlap in patients served with a Diagnosis Related Group (DRG) code for burns, trauma, and conditions of the nervous, respiratory, and circulatory systems. Table 2 also includes the baseline C-statistic of the model. The model considered is the LACE+ Index [24] which predicts unplanned 30-day readmission or mortality. We collected the highest LACE+ prediction during each index admission (i.e., not an unplanned readmission) for adult patients at each site between August 2021 and December 2024 and whether the patient experienced an unplanned readmission in the 30 days following discharge. Table 2 also provides detailed information about the temporal distribution of observations at site.

#### 2.1.2. Experimental methodology

Since we collected model predictions and outcomes from 2021 to 2024, we perform our evaluation temporally. For example, the experiment labeled “21 →22” uses data from 2021 for calibration and data from 2022 for validation. Using the previously defined framework, we consider four model recalibration strategies. First, we consider model recalibration pre-localization using the calibrator derived from the main site, $H_{0}$, (i.e., the model $l_{0}(f(x))$, denoted as “Main Site Calibrator”) and the calibrator derived from aggregating all data (i.e., the model $l_{A}(f(x))$, denoted as “Combined Calibrator”). These two strategies represent scenarios where a model has been tuned to the health system prior to deployment but has not been localized for each site. We then consider two localization strategies. The first generates local calibrators $l_{i}(f(x))$ using only the available data at each site in each training period (denoted as “Localization with Available Data”). The second oversamples available data at each site to meet the minimum sample size determined by Riley's sample size guidelines for models with a binary outcome [11]. All calibrator models are implemented using Platt's method [22]. Since our prediction task is binary, we use the C-statistic to measure overall model discrimination. We calculate the calibration intercept α and slope β where ideal values are α=0 and β=1, respectively.

### 2.2. Experiment 2: Simulation of data augmentation to mitigate sample size concerns.

To characterize the efficacy of data augmentation to mitigate sample size concerns, we developed a simulation using a publicly available critical care database that contains admissions across multiple sites nationwide [25]. We opted to use this external database because we did not have direct access to the all model inputs in the Real-World Evaluation. We assume a framework where a network of hospitals develops a machine learning model using data from the main site and then optimize the calibration of this model at the smaller community of hospitals within the network. In this experiment, we develop a model $l_{0}(f(x))$ using data from the main site $\left(H_{0}\right)$. We then evaluate the impact of developing calibrator models $l_{i}$ at each of the satellite hospitals $H_{i}$ using data augmentation techniques in the form of oversampling and synthetic data generation [17,18] to achieve necessary data volumes and compare these local calibrators to a global calibrator $l_{A}$ using data from all sites. For the remainder of this paper, we refer to this experiment as the Simulation Study.

#### 2.2.1. Data

We perform our analysis with data from the eICU Collaborative Research Database [25]. This database contains de-identified ICU admissions from 2014 and 2015 for patients at 208 sites across the US. Each site is affiliated with an aggregate indication of its number of beds (e.g., >500, 250–499, 100–249, <100) and US Census region (i.e., South, Northeast, Midwest, and West). Using this data, we consider two approaches to selecting sites for analysis. First, in the “Combination” approach, we combine individual sites based on size (e.g., all hospitals in each region with more than 500 beds are considered as the main site or $H_{0}$ for that region). This approach is designed to ensure ample representation from self-disclosed smaller sites, some of which may have <200 patients.

The second approach we consider for evaluating data augmentation in the multi-site scenario is to randomly select sites within each US census region and simulate model development and localization at these sites (denoted as the “Bootstrap” approach). To mirror the other evaluations considered so far, we select four sites from within a US census region with at least 500 training samples. The largest of the selected sites is designated as the primary site from which the model is developed, while the remaining sites are designated as the satellite sites. Tables C.9, C.12, C.15 in Appendix C list the available sites by ID and size in the training and test cohorts. Additionally, we focus the evaluation in the second approach to the impact of data augmentation on model localization.

For the simulation, we consider sites in the Northeast and South. In the southern US, there is a wide range in data volumes across hospital sizes; whereas, in the northeastern US, there is less variation in the number of patients by hospital size. Table 5 provides the size of each cohort based on hospital size and region. Patients with a discharge year of 2014 were partitioned into $T_{{\mathrm{train}}_{i}}$ and patients with a discharge year of 2015 were partitioned into $T_{{\mathrm{eval}}_{i}}$. The eICU database contains features necessary to compute APACHE IV (Acute Physiology, Age, and Chronic Health Evaluation) hospital mortality prediction score. Using the features included in this score, the prediction task for our simulation is mortality during ICU stay.

#### 2.2.2. Models

The classification model f in our simulation is a CatBoost gradient-boosted tree [26] trained to predict ICU mortality based on the APACHE features. This model is trained using $T_{{\mathrm{train}}_{0}}$ – patients from $H_{0}$ with a discharge year of 2014 (see Figure A.2). We use a random 75% of the data from $H_{0}$ in 2014 to train the gradient-boosted tree to serve as our clinical prediction model to mitigate the likelihood of overfitting. The remaining 25% of the patients from $H_{0}$ in 2014 are used to train the calibrator $l_{0}$. For sites $H_{1},\;H_{2}$, and $H_{3}$, we use all data from 2014 to train the calibrator $l_{1},\;l_{2}$, and $l_{3}$, respectively. The calibrator model $l_{i}$ is implemented using Platt's method [22]. For $H_{1},\;H_{2}$, and $H_{3}$, local calibrator models are trained on $T_{{\mathrm{train}}_{i}}$ – patients from $H_{i}$ with a discharge year of 2014 – with data augmentation if applicable. The aggregate calibrator model $l_{A}$ is trained on all available data across sites from 2014.

#### 2.2.3. Data augmentation

In the event that a site does not have the requisite number of samples for recalibration, we hypothesize that data augmentation in the form of oversampling or synthetic data generation may reduce this burden on smaller medical centers and facilitate updating to improve performance. We base our hypothesis on previous work by Jackson and colleagues [17] and Theodorou and colleagues [18], both of whom apply similar data augmentation techniques to improve the fairness of AI/ML-based clinical classifiers. Modern synthetic data generation is dependent on modeling the univariate and multivariate distributions per feature (e.g., Gaussian Copula [27]) or by deep neural networks such as generative adversarial networks (GANs) and variational autoencoders (VAE) [16,28]. GANs commonly consist of two deep neural networks: a generator network that generates plausible synthetic instances and a discriminator network that determines if a data point is artificially generated. Alternatively, VAEs are built upon the autoencoders, which attempt to reconstruct data instances by reducing the representation to a latent space and expanding the latent representation into the reconstruction. Variational autoencoders build upon this network type by using a probabilistic model in the latent representation to encourage generation of synthetic instances. We choose to use a conditional GAN [29] (CTGAN), Tabular VAE (TVAE) [29], and Gaussian Copula (GaussianCopula) [27] implemented in the Synthetic Data Vault for our analyses.

The computational requirements for the proposed synthetic data generation may be difficult for smaller medical centers to realize. Thus, we consider two possible scenarios to reflect the computational resources available to satellite sites with two strategies each per scenario (Figure A.2 and Table 3) [17,18]. In the first scenario (Figure A.2, Scenario 1), we assume the satellite hospitals ($H_{i}$, with i≥1) have access to the computational resources necessary to augment their data with synthetic instances. Let t be the number of additional observations needed to reach the $t_{i}$ observations required for training the local calibrator. In this scenario, the local site will train a synthetic data generation model on their original data. With local synthetic data generators trained, each local site could use either of the following strategies: (1) sample the t required instances needed from the synthetic data generator or (2) sample ⌈t/2⌉ observations from the synthetic data generator and oversample another ⌊t/2⌋ from $T_{{\mathrm{train}}_{i}}$ (sampling with replacement if necessary).

In the second scenario, we assume the satellite hospitals do not have access to the computational resources necessary to augment their data with synthetic instances (Figure A.2, Scenario 2). The first strategy for this scenario is that satellite hospitals simply oversample with replacement from $T_{{\mathrm{train}}_{i}}$ to reach the necessary number of samples. Alternatively, the satellite hospitals could provide the main hospital $\left(H_{0}\right)$ with access to $T_{{\mathrm{train}}_{i}}$. The main hospital could then develop a common synthetic data generator based on data from the satellite hospitals with each site receiving t synthetic observations from the generator conditioned on the respective site.

#### 2.2.4. Experimental methodology

We run the proposed simulation five times. Within each execution of the simulation, each data augmentation and respective recalibration is performed five times. This results in 25 measurements of calibration performance for each approach, scenario and sampling method. For the Bootstrap approach, each iteration of the simulation results in a new selection of four random sites.

Since our prediction task is binary, we use the C-statistic to measure overall model discrimination and measure calibration with the calibration intercept α and slope β, where ideal values are α=0 and β=1, respectively. Since we use Platt's Scaling for recalibration, C-statistic will not change when the model is recalibrated. To streamline results for Experiment 2, we report the Euclidean distance between the ideal calibration point of (0,1) and the observed point (α,β), calculated as $\sqrt{(\alpha -0{)}^{2}+(\beta -1{)}^{2}}$.

## 3. Results

### 3.1. Ability of satellite hospitals to update clinical prediction models

The Real-World Evaluation assesses the discrimination and calibration of the LACE+ Index at a network of hospitals in the Middle Tennessee area. These hospitals include one urban academic medical center and two satellite hospitals in predominantly rural areas. Table 2 presents the C-statistic and Fig. 1 presents the coefficients α and β of the calibration line of the model developed using data from the main site $H_{0}$ and evaluated at all three sites $H_{0},\;H_{1}$, and $H_{2}$. We compute the 95% confidence intervals based on the performance at three evaluation scenarios across a total of four years. We note that the C-statistic, which indicates the model's overall ability at identifying the outcome of interest, differs by as much as about 12% across the individual sites and $H_{0}$ does not have the best discrimination.

Model calibration, which is measured by the slope β and intercept α of the calibration line formed by the model's predictions, exhibits more noticeable differences across the sites. When using a calibrator derived from the main site (Fig. 1, panel A), we observed that the satellite sites experienced worse calibration than the primary urban site. When we investigate the number of samples available at each site and the number of samples required for localization (Table 4), it can be seen that neither satellite site has the requisite samples for proper recalibration. As a result, when models are localized using only the data available locally (i.e., $T_{{\mathrm{train}}_{i}}$ in Fig. 1, Panel C), it can be seen that while calibration moves closer to the ideal, variation in performance also increases beyond what is seen when using the calibrator at the main site (Fig. 1, Panel A).

In response to this increased variance, we considered two strategies to improve sample sizes locally. The first strategy was to simply combine the available data at all sites for the given time period (i.e., ${⋃}_{i=0}^{n}\;T_{{\mathrm{train}}_{i}}$). While combining all available data across all sites does ensure that each site has the requisite number of samples, this approach resulted in calibration very closely aligned to using the calibrator derived from the main site, $l_{0}$. This is likely due to the fact that samples from site $H_{0}$ composes between 88% and 97% of available samples for recalibration for each year in which the data was available. This results in satellite sites $H_{1}$ and $H_{2}$ having a low prevalence among samples used for recalibration. Ultimately, this indicates that global recalibration may be ill-suited when patients at satellite sites that have low prevalence in the overall sample space for recalibration.

We also considered localization by training site-specific calibrators, $l_{1}$ and $l_{2}$, using oversampling (OS) of available data to reach minimum sample size requirements (Fig. 1, Panel D). We find that for site $H_{1}$, oversampling to reach sample size requirements was sufficient to successfully train the local calibrator $l_{1}$. For site $H_{2}$, oversampling available data does not result in the same improvement in calibration as seen by $H_{1}$. We suspect this is a result of the fact that $H_{2}$ consists of less than 3% of all samples — the smallest site in our evaluated cohort. Thus, it is reasonable that the vast amount of oversampling required to reach minimum sample size requirements ultimately caused the calibrator to overfit.

### 3.2. Data augmentation to mitigate sample size concerns

Given the need for larger sample sizes to enable model localization at satellite facilities, we explored synthetic data generation (SDG) as an alternative to oversampling, which our initial findings in the Real World Evaluation indicated may be insufficient for recalibration. Tables C.9, C.12, and C.15 in Appendix C highlights the distribution of samples in the eICU database by hospital site. As described above, the combined approach merged sites within the same size category to emulate a multi-site network of hospitals and robustness of the SDG strategy was evaluated with a bootstrapping approach that samples sites with at least 500 training samples.

#### 3.2.1. Combined results

We note in Table 5 that the northeast US has fewer patients in this database compared to the southern US. One satellite site in the southern US and all three satellite sites in the northeast US required data augmentation to reach the minimum sample size for recalibration.

Table 5 presents the C-statistic and 95% confidence interval when $H_{0}$ trains and calibrates a clinical prediction model on $T_{{\mathrm{train}}_{0}}$ and provides this model to $H_{1},\;H_{2}$, and $H_{3}$ without local recalibration. There were minor differences in C-statistics across sites in both regions; however, we note moderate variability in calibration across different sites. For sites in the southern US, only $H_{3}$ requires data augmentation to meet sample size requirements for recalibration, but for the northeast US, all three satellite sites $H_{1},\;H_{2}$, and $H_{3}$ require data augmentation. We note that generating each of these sites required combining data from hospitals of similar sizes within each region into $H_{0},\;H_{1},\;H_{2}$, and $H_{3}$.

Table 6 (full results in Appendix D) presents the Euclidean distance between the point obtained from the observed calibration intercept and slope (α,β) and the ideal calibration intercept and slope of (0,1).

For the APACHE model in the southern US, sites $H_{1}$ and $H_{2}$ had the requisite samples for recalibration, and we found that using locally available data for these sites yielded the best calibration on unseen data. Of note, $H_{2}$ performance displayed higher variance than $H_{1}$ and has a smaller number of available instances for recalibration and evaluation. This could be a result of an overall smaller pool of data used to evaluate calibration at this site. Only $H_{3}$ required data augmentation to reach sample size requirements and, in this situation, we observed that training CTGAN on data from all sites then conditionally sampling to retrieve synthetic instances for $H_{3}$ yielded the best calibrated predictions across data augmentation strategies.

For the APACHE model in the northeast US, we find that all three satellite sites, $H_{1},\;H_{2}$, and $H_{3}$ required data augmentation to meet the sample size requirements for recalibration. For sites $H_{1}$ and $H_{2}$, oversampling or using locally available data resulted in the best performance, with conditionally sampled TVAE-generated data resulting in the best calibrator using synthetic data generation. For $H_{3}$, the combination approach using synthetically generated data and oversampled local data produced the best local calibrator.

Both geographic regions had differing capabilities at the primary site $H_{0}$. In the southern US, the main site had the requisite samples for recalibration; however, calibration performance using the main site calibrator $l_{0}$ had higher variation than the combined calibrator $l_{A}$. For the northeast US, the main site did not have the requisite number of samples for local recalibration; however, we see that combining synthetically generated data and oversampling the existing data resulted in a better calibrated classifier than the combined calibrator $l_{A}$.

#### 3.2.2. Bootstrap results

To better understand the generalizability of SDG as an augmentation approach for model localization, we also considered a scenario in which we considered sites with at least 500 training samples (see Appendix C). Table 7 (full results in Appendix D) the Euclidean distance between the point obtained from the observed calibration intercept and slope (α,β) and the ideal calibration intercept and slope of (0,1). In the northeast US, for sites $H_{1}$ and $H_{2}$, most optimally calibrated predictors were derived using data generated from a variational autoencoder trained on data from all considered sites (i.e., $H_{0},\;H_{1},\;H_{2},\;H_{3}$). Site $H_{0}$ had the best calibrated predictors when using all available data without synthetic data augmentation, whereas site $H_{3}$ achieved the best calibrated predictions using locally available data to derive predictors. In the southern US, the predictions for sites $H_{0}$ and $H_{1}$ were best calibrated using the variational autoencoder, whereas site $H_{3}$ had the most success using CT-GAN to generate synthetic instances. Site $H_{2}$ was the only site to achieve optimal calibration when deriving a predictor using locally available data.

Similar to when sites were combined, the deep learning techniques for synthetic data augmentation (i.e., CT-GAN or variational autoencoders) were the data augmentation techniques that resulted in the best calibrated predictive model. By contrast, predictive models developed using the Gaussian copula to augment training data tended to exhibit the worst calibration across all scenarios. Additionally, both regions display comparable amounts of variation in calibration performance, likely due to comparable sizes of individual sites within each region.

## 4. Discussion

### 4.1. Real-world evaluation

We evaluated the ability of smaller sites in a multi-site hospital network to localize clinical prediction models for optimal reliability on their patient population. In our evaluation, one site had vastly more data available for localization, while the other two sites had much smaller patient populations that could not meet localization guidelines [11]. We believe this heterogeneity and imbalance in the number of examples per site lead to the inconsistent ability of local sites to optimize prediction models for their respective populations. It is event that future work with additional models, as well as additional multi-hospital networks, is necessary to determine the relationship between site imbalance on model localization. Such insights could provide guidance on how to coordinate localization across networked sites to effectively leverage system-wide information and serve patients regardless of where they access care.

Additionally, our analysis illustrates that there were mild differences in discrimination and moderate deterioration in calibration when applying a model developed at a larger primary site to smaller satellite sites. This is not unexpected in the context of previous studies [8,9,14]. Austin and colleagues [14] developed validation techniques of prediction models across multiple sites. In their evaluation of a mortality prediction model for heart failure patients, they noticed more pronounced differences in C-statistic than in our study; however, we noticed more variation across sites in both calibration slope and intercept than their study. Additionally, Lyons and colleagues [30] measured the discrimination of a proprietary sepsis prediction model in a network of 9 hospitals and demonstrating wide variations in performance. Given the differences in C-statistic and calibration performance in both our study and others, we hypothesize this will be a pronounced issue for clinical prediction models applied at multiple sites, even within healthcare system networks. This will require protocols for rigorous external validation across multiple networks, sites, and model application scenarios. We also note that our findings are consistent with prior work that indicates calibration is more sensitive to differences between training and validation cohorts than discrimination [8,9].

Our results, however, highlight a potential disparity between rural and urban patients that is commonly overlooked in evaluations of AI for healthcare use [31,32]. A recent literature review noted that performance differences in AI between rural and urban sites was underwhelming [31] and, when evaluated, rural populations often suffered from lower algorithmic performance compared to urban populations for both new and previously developed models [32–35]. Further investigation into this disparity is needed which includes understanding the scope of the disparity at the network, state, and national level, developing and evaluating localization techniques that account for geography, and improving algorithmovigillance [36] and algorithmic auditing [37] to accommodate differences in various geographic populations.

### 4.2. Simulation study

In light of concerns posed when evaluating calibration of models across geographic sites, we performed a simulation study with a publicly available multi-site ICU database. We evaluated the efficacy of synthetic data generation using sites in the northeast and southern US. We find that for the southern US, deep learning-based approaches to generate sufficient data for local recalibration resulted in classifiers that were closest to an ideal calibration; however, for the northeast US, using existing data and oversampling was the most advantageous for two of the three satellite sites. We note for this simulation scenario there is generally less available data than for the APACHE model in the northeastern US. Thus, we posit that this lack of data could result in the synthetic data generators not producing adequate data for recalibration, and as a result recalibration efforts benefiting more from a combined approach of synthetic data and oversampling. A site with more available samples may benefit most from SDG as there is sufficient information to train the data generation process. For smaller sites with more limited data volumes, oversampling of existing data may outperform weakly trained or overfit SDG. The deep learning approaches may have overfit the data in the northeast US, resulting in less robust synthetic instances. This hypothesis is further validated by Yan et al. [16] who found that using deep learning approaches on samples and concepts with low prevalence may result in statistical properties being inadequately learned by GAN-based synthetic data generation techniques. However, we also observed that in some cases combining data across sites within a health system may enable SDG to produce additional samples for recalibration when the main site itself does not have the requisite samples for recalibration, as we observed for the northeast US (Tables 6 and 7). Further research is needed to refine these observations and better characterize when oversampling or SDG should be applied, provide sites with methods to estimate sample size requirements for SDG, and develop actionable data augmentation guidelines.

Despite concerns about smaller sample sizes used to train synthetic data generation techniques, we found deep learning-based approaches typically produced better samples for recalibration than the statistical synthetic data generation technique tested. We further observe that recalibration was more successful using data generated by deep learning-based approaches when generator models were trained on all available data as opposed to at individual sites (e.g., conditional sampling approach). This may be a promising approach as small health centers may not have the computational capabilities or technical staff to facilitate local SDG [38–40]. Research is needed to improve the accessibility of these technologies to lower-resourced facilities, but the conditional sampling approach can be one method to help alleviate these concerns. Through collaborations and partnerships between smaller, less-resourced medical centers and larger medical centers with more computational resources, it could be possible to facilitate scenarios in which the site with the most computational resources producing synthetic data would reduce the technical and staffing burdens on lower-resourced sites; however, there are also potential concerns regarding data access and privacy. In our study, we assumed that the primary hospital had the right to access data from the satellite hospitals under the HIPAA privacy rule [41]. If, however, this is not the case, additional approaches such as federated learning [42] and federated synthetic data generation [43] would need to be considered to help mitigate these privacy concerns while providing the benefits of collaborative data generation.

We acknowledge that there remain other scenarios in which the proposed strategies could fail. Some community hospitals are not associated with a larger network or other type of consortium, or they are associated with smaller networks without access to the computational infrastructure necessary to perform synthetic data generation. In these situations, further work would be needed to ensure equitable access to synthetic data generation or other methods of data augmentation.

Finally, guidelines for minimum sample size for estimating recalibration parameters also apply to determining the number of samples necessary to perform validation of model discrimination and calibration. Since the southern US had a higher data volume than the northeast US, the southern sites had the minimum sample size for recalibration and validation. The northeast US, however, did not meet the sample size requirements for robust validation (see Table 5). Thus, another possible reason as to why synthetic data generation was not the best data augmentation technique could be due to an insufficient data volume for validation to provide a complete picture of model calibration and discrimination. Performing data augmentation for validation was outside of the scope of this work and requires additional considerations to prevent data leakage and evaluate whether generated data are robust for model validation.

### 4.3. Limitations

There are several limitations that we wish to highlight, which provide future work directions that could enhance the robustness and interpretation of our findings. First, we only consider the scenario in which a machine learning model is trained using main site data. Future work should also consider the scenario with a model trained across all sites and other validation techniques for multi-site models [14]. We focused on synthetic data generation with little fine-tuning, and we did not use synthetic data generation methods developed specifically for generating synthetic EHR observations. In our simulation study, we combined sites based on number of beds and used bootstrapping to vary which sites were used in analysis. However, we cannot completely analyze the impact of the specific geographic (e.g., state, county, ZIP code) location on model calibration and localization in these data. Thus, we believe that future investigations should include data sources that have multiple sites with geographic information, such as the All of Us Research Program [44] or other multi-center hospital networks. We primarily focused on data augmentation; however, other strategies such as model distillation may also be advantageous for satellite sites. While we focused primarily on model calibration, Davis and colleagues [45] demonstrated algorithmic fairness can drift over time, and we suspect fairness drift may also occur geographically. This study presents additional evidence regarding potential disparities in performance between rural and urban sites in a healthcare system [32–35]. Future investigations should consider the impact of localization schemes and data augmentation on algorithmic fairness across sites with networked healthcare systems and the potential for fairness drift across sites [45]. Finally, while our work considers the impact of insufficient data volume for model transportability further investigation is needed into the scenario when the validation cohort does not meet sample size requirements.

## 5. Conclusion

In this work, we explore the transportability of clinical prediction models across geographically diverse sites and the ability of smaller, satellite healthcare centers to locally recalibrate models given available data resources. Through our analysis, we see that institutional connections to large medical centers are not enough to promote accurate prediction models at all sites within a healthcare system and data augmentation, including synthetic data generation, may provide the necessary data volumes to enable local recalibration at smaller facilities. Data augmentation approaches, however, may assume unrestricted access to data from across affiliated healthcare facilities or local access to computational resources that may be infeasible for some healthcare networks. Addressing these needs will require novel approaches that balance local and network-supported data augmentation to promote CPM performance and deliver reliable, equitable predictive insights to all communities regardless of where they access care.

## Supplementary Material

Supplementary Material

## Figures and Tables

**Fig. 1.**
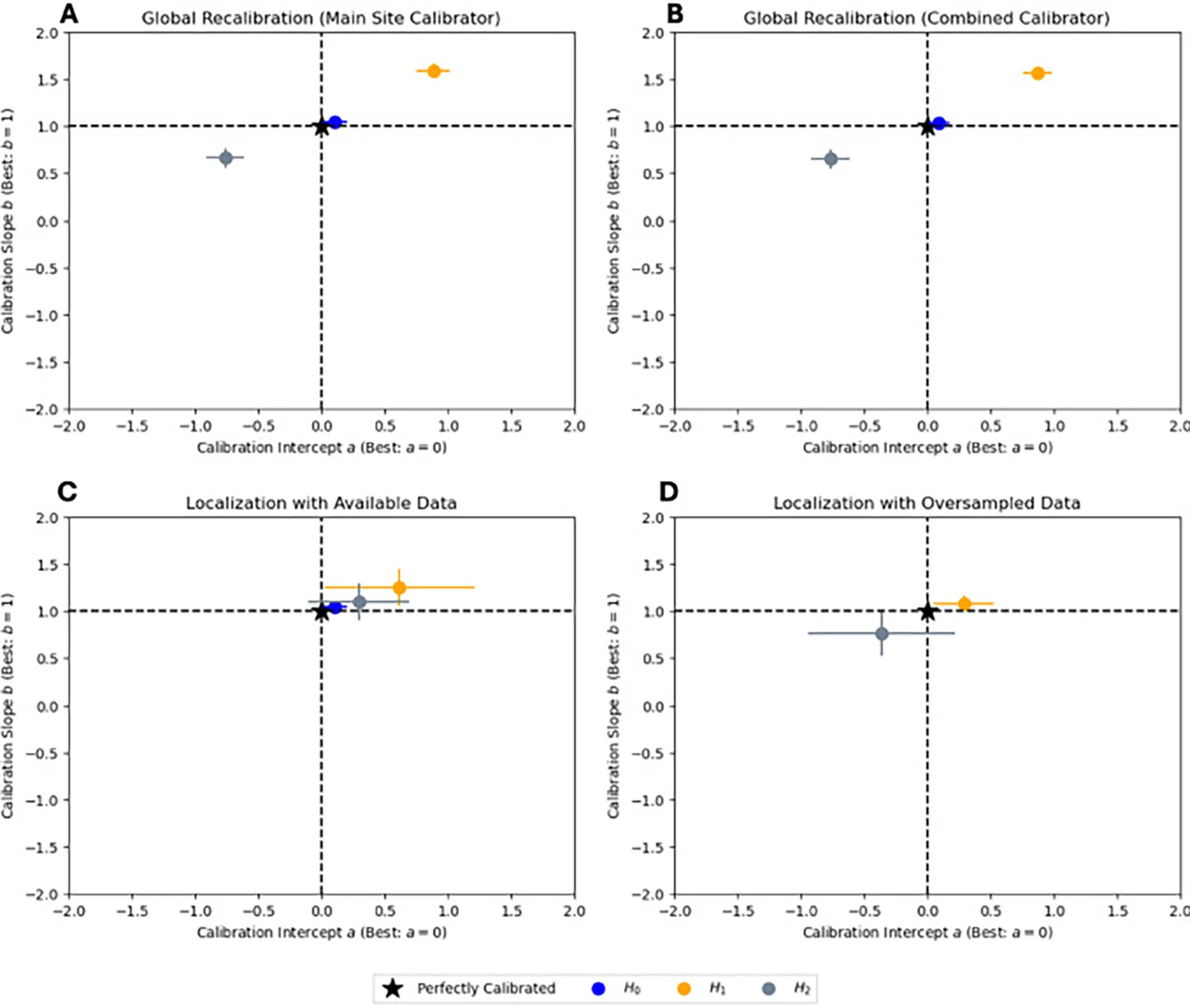
Plots of slope/intercept of the calibration curve for each data augmentation method. Note how synthetic data generation approaches pushes calibration of the desired model to the ideal point (0,1), denoted by a star.

**Table 1:** Mathematical formalization of project.

Variable	Definition

$H_{0}$	Main hospital or academic medical center associated with a network of hospitals
$H_{1},H_{2},\ldots ,H_{n}$	Satellite hospitals associated with the care of local communities
$T_{{\mathrm{train}}_{i}}$	The set of patients at $H_{i}$ during the model’s training period
$T_{{\mathrm{eval}}_{i}}$	The set of patients at $H_{i}$ during the model’s evaluation period
$t_{i}$	The number of additional samples determined to be necessary to augment $T_{{\mathrm{train}}_{i}}$ to produce a properly calibrated model at $H_{i}$ [11,12]
x	Individual patient
m(x)	Model prediction for x
f(x)	Machine learning algorithm producing logit of the likelihood of the event of interest
l(f(x))	Logistic regression model calibrating logit to probability of event of interest
$l_{i}$	Logistic regression calibrator derived from data from $T_{{\mathrm{train}}_{i}}$
$l_{A}$	Logistic regression alibrator derived from combining the training data from all sites (e.g., ${⋃}_{i=0}^{n}\;T_{{\mathrm{train}}_{i}}$)

**Table 2:** Site information including generalized, county-level area deprivation Index (ADI) and population density (in people per square mile) to prevent identifiability of the sites, eligibility for HRSA rural health funding, and baseline model C-statistic before recalibration.

Site	Population density	ADI	HRSA RH funding eligible	Model C-Statistic	Number of predictions			
					2021	2022	2023	2024
$H_{0}$	>1200	<100	No	0.63 (0.63, 0.63)	42,034	41,278	43,596	46,135
$H_{1}$	<250	>100	Yes	0.68 (0.68, 0.68)	1218	3998	4346	4673
$H_{2}$	<250	>100	Yes	0.56 (0.56, 0.56)	275	1151	1289	1584

A higher ADI indicates higher disadvantage. Note: RH = Rural Health.

**Table 3:** Description of data augmentation methods in the Simulation Study.

	Name	Description
Scenario 1: Local sites have computational resources supporting synthetic data generation	SDG	Train a synthetic data generator on $T_{{\mathrm{train}}_{i}}$. Generate *t* instances required to reach minimum sample size requirements
	SDG + Oversample	Train a synthetic data generator on $T_{{\mathrm{train}}_{i}}$. Generate *t*/2 instances from SDG. Oversample $T_{{\mathrm{train}}_{i}}$ with replacement for the remaining *t*/2 instances.
Scenario 2: Local sites lack computational resources supporting synthetic data generation	Oversample	Oversample $T_{{\mathrm{train}}_{i}}$ with replacement to reach the minimum sample size required.
	SDG, Conditional	Train a synthetic generator on $\cup_{i=0}^{n}T_{{\mathrm{train}}_{i}}$. Sample *t* total instances with the desired site.

Note: SDG = Synthetic Data Generation.

**Table 4:** Sample size requirements for each site in the Real-World Evaluation, aggregated across time.

Site	Samples required (95% CI)	Samples available (95% CI)	#RG (95% CI)
$H_{0}$	14 244 (14 224, 14 263)	42 303 (42 260, 42 345)	−28 059 (−28 121, −27 997)
$H_{1}$	16 461 (16 350, 16 572)	3187 (3125, 3249)	13 274 (13 103, 13 445)
$H_{2}$	15 299 (15 234, 15 364)	905 (885, 925)	14 394 (14 315, 14 474)

Number of samples required to generate is denoted #RG.

**Table 5:** C-statistic and 95% confidence interval of model on unseen data in the Simulation Study.

	Site	C (95% CI)	*α* (95% CI)	*β* (95% CI)	Samples required	Samples available	Number of predictions	
							2014	2015
South	$H_{0}$	0.85 (0.85, 0.85)	−0.38 (−0.48, −0.28)	0.79 (0.76, 0.81)	6007	8810	20 144	23 344
	$H_{1}$	0.85 (0.85, 0.85)	−0.45 (−0.53, −0.37)	0.79 (0.77, 0.81)	6961	12 952	12 952	15 330
	$H_{2}$	0.84 (0.84, 0.84)	−0.41 (−0.47, −0.35)	0.85 (0.83, 0.86)	6854	9074	9074	11 492
	$H_{3}$	0.84 (0.84, 0.84)	−0.23 (−0.35, −0.11)	0.84 (0.81, 0.87)	5787	3242	3242	5840
Northeast	$H_{0}$	0.81 (0.81, 0.82)	−0.57 (−0.58, −0.56)	0.57 (0.57, 0.58)	3395	2586	6040	6042
	$H_{1}$	0.81 (0.81, 0.82)	−0.84 (−0.85, −0.84)	0.51 (0.5, 0.51)	3555	1302	1842	2024
	$H_{2}$	0.81 (0.8, 0.82)	−1.09 (−1.18, −1)	0.62 (0.6, 0.65)	4309	1842	1302	1258
	$H_{3}$	0.79 (0.79, 0.8)	−0.87 (−0.92, −0.82)	0.54 (0.53, 0.56)	4645	1158	1158	1136

Calibrator used is the main site calibrator, $l_{0}$. Sample size requirements for each site in the Simulation Study, aggregated across time. Rows in bold indicate sites in which data augmentation is necessary. Number of samples required to generate is denoted #RG.

**Table 6:** Results for eICU (North and Southeast, Midwest and West in Appendix).

		$H_{0}$	$H_{1}$	$H_{2}$	$H_{3}$

Northeast	Locally Available Data	0.62 (0.56, 0.69)	0.21 (0.18, 0.25)	0.20 (0.15, 0.24)	0.50 (0.42, 0.58)
	Combined Calibrator	0.24 (0.21, 0.27)	0.51 (0.48, 0.53)	0.61 (0.55, 0.67)	0.60 (0.54, 0.67)
	Main Site Calibrator	0.62 (0.56, 0.69)	0.90 (0.85, 0.94)	1.07 (0.99, 1.14)	0.98 (0.91, 1.06)
	Oversampled Data	0.62 (0.56, 0.68)	0.23 (0.19, 0.27)	0.22 (0.16, 0.27)	0.52 (0.45, 0.60)
	GaussianCopula, Conditional	0.29 (0.25, 0.33)	1.19 (1.11, 1.26)	1.41 (1.27, 1.56)	2.40 (2.17, 2.63)
	CTGAN, Conditional	0.44 (0.37, 0.52)	0.65 (0.47, 0.82)	0.76 (0.57, 0.96)	0.53 (0.36, 0.71)
	TVAE, Conditional	0.52 (0.46, 0.58)	0.32 (0.23, 0.41)	0.31 (0.22, 0.40)	1.14 (1.03, 1.25)

South	Locally Available Data	0.36 (0.31, 0.41)	0.11 (0.08, 0.14)	0.27 (0.19, 0.34)	0.77 (0.70, 0.84)
	Combined Calibrator	0.10 (0.08, 0.12)	0.19 (0.16, 0.21)	0.32 (0.26, 0.38)	0.16 (0.12, 0.21)
	Main Site Calibrator	0.36 (0.31, 0.41)	0.47 (0.42, 0.51)	0.59 (0.51, 0.67)	0.20 (0.15, 0.25)
	Oversampled Data	–	–	–	0.78 (0.69, 0.87)
	GaussianCopula, Conditional	–	–	–	1.09 (0.99, 1.18)
	CTGAN, Conditional	–	–	–	0.39 (0.27, 0.50)
	TVAE, Conditional	–	–	–	0.70 (0.58, 0.81)

“–” indicates that the site had the requisite samples and did not require data augmentation.

**Table 7:** Results for eICU bootstrapped experiments (North and Southeast, Midwest and West in Appendix).

		$H_{0}$	$H_{1}$	$H_{2}$	$H_{3}$

Northeast	Locally Available Data	0.59 (0.52, 0.67)	0.38 (0.29, 0.47)	0.78 (0.74, 0.83)	0.53 (0.41, 0.65)
	Combined Calibrator	0.20 (0.17, 0.23)	0.32 (0.29, 0.35)	0.79 (0.74, 0.85)	0.58 (0.46, 0.69)
	Main Site Calibrator	0.59 (0.52, 0.67)	0.89 (0.81, 0.97)	1.27 (1.19, 1.34)	0.81 (0.56, 1.06)
	Oversampled Data	0.61 (0.52, 0.70)	0.39 (0.30, 0.48)	0.78 (0.73, 0.83)	0.57 (0.45, 0.69)
	GaussianCopula Conditional	0.53 (0.40, 0.66)	0.50 (0.42, 0.58)	0.86 (0.68, 1.03)	2.77 (2.18, 3.35)
	CTGAN Conditional	0.71 (0.62, 0.80)	0.51 (0.42, 0.60)	0.95 (0.84, 1.07)	0.78 (0.63, 0.93)
	TVAE Conditional	0.43 (0.35, 0.51)	0.31 (0.24, 0.38)	0.70 (0.59, 0.81)	0.65 (0.45, 0.85)

South	Locally Available Data	1.16 (0.91, 1.40)	0.32 (0.23, 0.41)	0.84 (0.62, 1.05)	0.58 (0.43, 0.73)
	Combined Calibrator	0.94 (0.65, 1.24)	0.85 (0.51, 1.20)	0.72 (0.48, 0.95)	0.67 (0.47, 0.88)
	Main Site Calibrator	1.16 (0.91, 1.40)	1.30 (1.11, 1.49)	1.33 (1.15, 1.51)	1.52 (1.34, 1.69)
	Oversampled Data	1.19 (0.92, 1.46)	0.37 (0.26, 0.48)	0.79 (0.61, 0.98)	0.59 (0.42, 0.76)
	GaussianCopula Conditional	2.49 (0.80, 4.18)	2.21 (1.52, 2.91)	2.35 (1.35, 3.35)	2.87 (2.06, 3.68)
	CTGAN Conditional	1.97 (0.87, 3.07)	1.15 (0.64, 1.67)	0.86 (0.65, 1.08)	1.82 (1.26, 2.39)
	TVAE Conditional	1.53 (0.92, 2.14)	0.74 (0.59, 0.88)	0.62 (0.49, 0.75)	1.17 (0.82, 1.53)

“–” indicates that the site had the requisite samples and did not require data augmentation.

## Data Availability

Data will be made available on request.

## Appendix A. Supplementary data

Supplementary material related to this article can be found online at https://doi.org/10.1016/j.jbi.2026.105012.
