# Supplementary material for: Community medical centers struggle to produce well-calibrated clinical prediction models: Data augmentation can help
Source: J Biomed Inform. Author manuscript; Available in PMC 2026 Oct 1. (PMC13629187; doi:10.1016/j.jbi.2026.105012)
Supplement: Supplementary file 1 [file NIHMS2208946-supplement-Supplementary_Material.pdf]

Appendix A. Additional Narrative Description of Framework

To formalize the scenario considered in this work, let us assume hospital H_0 represents the main hospital or academic medical center associated with a network of hospitals, while hospitals $H_1, H_2, \ldots, H_n$ are satellite hospitals focused on the care of local communities (see Figure A.2). Let T_{train_i} represent the set of patients at H_i during the model's training period and T_{eval_i} be the set of patients at H_i during the model's evaluation period. Let m be the model trained on T_{train_0}. Let t_i represent the number of additional samples determined to be necessary to augment T_{train_i} to produce a proper calibrator model at H_i.

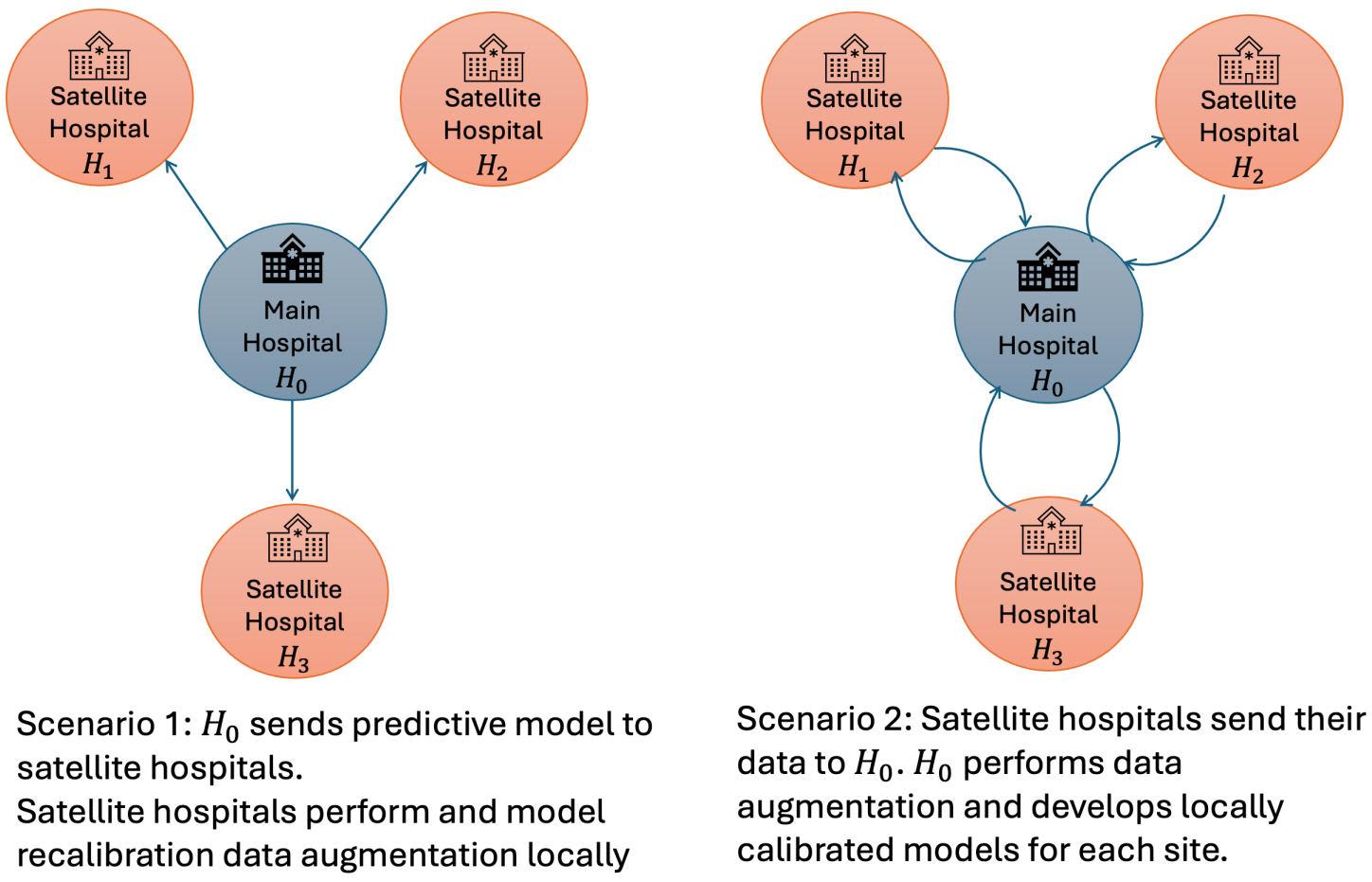

Figure A.2: Schematic depicting the scenarios for synthetic data generation.

The model's prediction for a patient x, denoted a $m(x)$, can be further considered as the composition of $l(f(x))$. In this composition, f denotes a machine learning algorithm, such as a gradient-boosted tree or neural network, trained to produce a value corresponding to the logit of the likelihood of the event of interest. This logit is then provided to a calibrator model $l(\cdot)$ that produces the calibrated probability of the event of interest. Commonly, l is a logistic regression model. While the machine learning model f remains static after initial training, we can compose different calibrator models depending on the data used. For example, a calibrator derived from data from T_{train_i} (e.g., H_i) will be denoted as $l_i(\cdot)$, and a calibrator derived from combining the training data from all sites (e.g., $\bigcup_{i=0}^{n} T_{train_i}$ will be denoted as $l_A(\cdot)$. Riley et al. [11, 12] derived several guidelines to determine the sample sizes needed to estimate recalibration parameters l_i.

Appendix B. Additional Site Information for the Real-World Experiment

Table B.8: Table 1. Distribution of overlap in selected DRG codes between Vanderbilt University Hospital and Vanderbilt's regional hospitals. Higher percentages indicate higher capability of regional hospitals.

**DRG Code**	**Disease Group Speciality**	**% Capability Overlap**	
		H_1	H_2
020-103	Nervous System	<1	15.4
163-208	Respiratory System	8.9	30.4
209-320	Circulatory System	4.4	17.8
927-935	Burns	<1	<1
955-965	Trauma	<1	<1

Appendix C. Additional eICU Dataset Information

Table C.9: Additional eICU Site Information for Simulation Experiments. If Size Category is N/A, the site is not included in the Combination evaluation approach. If 2014 column contains a value less than 500, the site is not included in the Bootstrap evaluation approach.

Table C.10: USA South

Site ID	Size Category	2014	2015
110	100 - 249	1584	1650
122	≥ 500	2600	2850
140	100 - 249	536	524
141	250 - 499	1490	1424
142	≥ 500	1482	1256
144	100 - 249	718	668
188	≥ 500	3288	3404
194	100 - 249	140	496
195	250 - 499	654	1576
196	<100	78	596
197	N/A	1566	1770
198	≥ 500	456	752
207	<100	168	272
208	≥ 500	2688	3012
210	100 - 249	484	518
215	100 - 249	608	628
217	250 - 499	1084	1076
220	250 - 499	696	682
224	100 - 249	472	480
243	≥ 500	2692	2732
244	100 - 249	1076	1098
245	100 - 249	580	582
301	100 - 249	950	968
307	250 - 499	1600	1740
312	100 - 249	582	610
318	250 - 499	1020	1132
345	250 - 499	1856	1536
417	250 - 499	1768	1668
429	N/A	300	250
433	N/A	136	98
434	<100	372	330
435	250 - 499	1364	1322
436	100 - 249	262	316
437	<100	48	56
438	N/A	166	178
439	100 - 249	296	344
440	250 - 499	1224	1130
443	≥ 500	3128	3452
458	≥ 500	3476	3260
459	100 - 249	536	616

Table C.11: USA Northeast

Site ID	Size Category	2014	2015
199	≥ 500	2246	2294
200	100 - 249	388	362
202	100 - 249	754	934
204	100 - 249	158	134
206	250 - 499	836	814
419	250 - 499	466	444
420	≥ 500	3794	3748
421	<100	536	572
422	100 - 249	304	316
423	<100	198	212
424	<100	424	352
425	100 - 249	238	278

Table C.12: Additional eICU Site Information for Simulation Experiments. If Size Category is N/A, the site is not included in the Combination evaluation approach. If 2014 column contains a value less than 500, the site is not included in the Bootstrap evaluation approach.

Table C.13: USA Midwest

Site ID	Size Category	2014	2015
56	<100	125	200
58	100 - 249	130	191
59	<100	311	543
60	<100	147	311
61	<100	86	147
63	100 - 249	679	1147
66	100 - 249	394	608
67	N/A	180	316
68	<100	243	449
69	<100	258	439
71	100 - 249	444	577
73	$\geq$ 500	3100	3959
79	$\geq$ 500	657	656
84	<100	24	20
86	N/A	9	6
90	<100	6	9
91	N/A	8	7
92	250 - 499	290	296
93	N/A	24	32
94	100 - 249	4	5
95	100 - 249	124	132
108	250 - 499	170	184
138	<100	85	91
248	100 - 249	812	866
249	<100	181	177
250	100 - 249	108	114
251	<100	152	166
252	$\geq$ 500	1688	1683
253	100 - 249	243	221
254	N/A	0	589
256	100 - 249	398	362
258	<100	174	175
259	250 - 499	244	342
262	<100	111	82

Table C.14: USA Midwest (Continued)

Site ID	Size Category	2014	2015
264	$\geq$ 500	2582	2655
265	N/A	0	33
266	N/A	117	116
267	<100	127	118
271	N/A	471	476
272	100 - 249	574	518
273	100 - 249	79	92
275	100 - 249	314	273
277	100 - 249	695	757
279	100 - 249	365	361
280	250 - 499	491	532
281	250 - 499	1195	1336
282	100 - 249	427	478
283	250 - 499	991	1106
300	$\geq$ 500	1765	1852
310	100 - 249	618	675
328	<100	277	248
336	100 - 249	438	544
338	$\geq$ 500	2245	2032
342	<100	176	79
353	100 - 249	310	385
355	<100	36	48
356	<100	32	34
357	N/A	382	363
358	100 - 249	165	195
360	100 - 249	67	219
361	<100	5	8
363	<100	41	29
364	100 - 249	150	179
365	N/A	872	724
416	$\geq$ 500	1034	1044
444	N/A	650	688
445	100 - 249	180	148
447	N/A	0	39
449	$\geq$ 500	1022	1088
452	N/A	454	420

Table C.15: Additional eICU Site Information for Simulation Experiments. If Size Category is N/A, the site is not included in the Combination evaluation approach. If 2014 column contains a value less than 500, the site is not included in the Bootstrap evaluation approach.

Table C.16: USA West

Site ID	Size Category	2014	2015
146	250 - 499	862	750
148	250 - 499	1452	1429
151	<100	0	14
152	100 - 249	940	807
154	250 - 499	954	921
155	100 - 249	475	377
156	<100	10	10
157	250 - 499	1056	1036
158	100 - 249	238	256
164	<100	92	85
165	≥ 500	1479	1574
167	≥ 500	2955	3137
171	100 - 249	1450	1357
174	<100	64	67
175	100 - 249	62	661
179	250 - 499	14	67
226	250 - 499	637	601
227	250 - 499	763	779
381	<100	96	129
383	100 - 249	230	271
384	250 - 499	466	518
386	100 - 249	248	279
387	≥ 500	0	589
388	100 - 249	541	451
389	<100	206	241
392	250 - 499	373	342
393	<100	61	66
394	250 - 499	983	954
396	100 - 249	295	280
398	<100	153	156
399	<100	135	120
400	250 - 499	754	759
402	<100	194	211
403	250 - 499	594	541
404	<100	209	199
405	100 - 249	166	201
407	100 - 249	351	340
408	<100	112	98
409	N/A	33	0
411	N/A	1742	1457
412	<100	158	150
413	N/A	1070	1341
414	N/A	0	54

Appendix D. Full Synthetic Data Generation Results

We evaluated three synthetic data generation (SDG) approaches (CTGAN, Variational Autoencoder, and Gaussian Copula) and three sampling strategies (SDG alone, SDG + oversample, conditional SDG). In most cases in which SDG was the most effective augmentation technique, deep learning SDG was more effective than statistical SDG (Gaussian Copula).

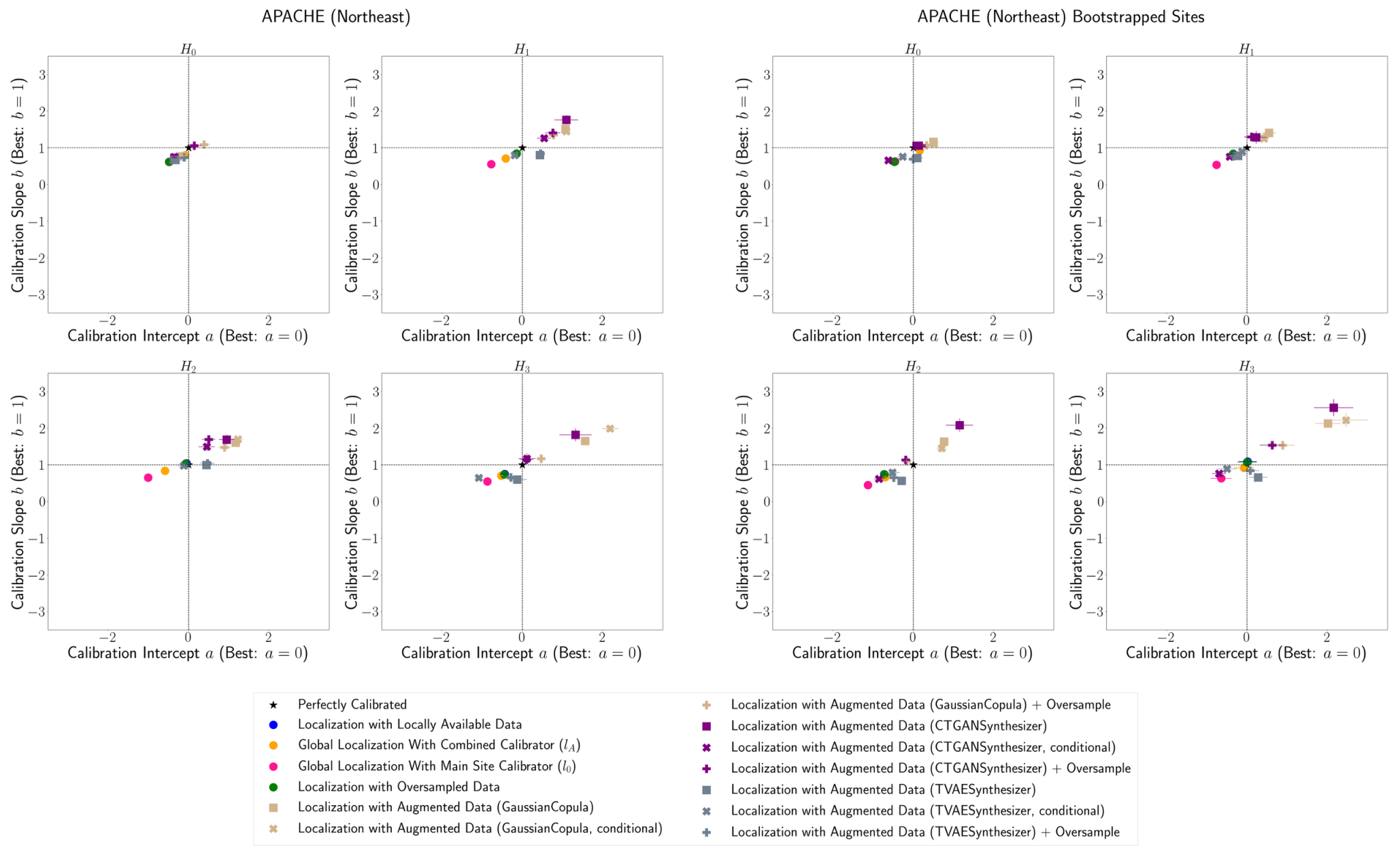

Figure D.3: Plots of slope/intercept of the calibration curve for each data augmentation method. Note how synthetic data generation approaches pushes calibration of the desired model to the ideal point (0,1), denoted by a star.

Table D.17: eICU Full Northeast Results (Combined)

	H_0	H_1	H_2	H_3
Locally Available Data	0.62 (0.56, 0.69)	0.21 (0.18, 0.25)	0.20 (0.15, 0.24)	0.50 (0.42, 0.58)
Combined Calibrator	0.24 (0.21, 0.27)	0.51 (0.48, 0.53)	0.61 (0.55, 0.67)	0.60 (0.54, 0.67)
Main Site Calibrator	0.62 (0.56, 0.69)	0.90 (0.85, 0.94)	1.07 (0.99, 1.14)	0.98 (0.91, 1.06)
Oversampled Data	0.62 (0.56, 0.68)	0.23 (0.19, 0.27)	0.22 (0.16, 0.27)	0.52 (0.45, 0.60)
GaussianCopula	0.33 (0.27, 0.39)	1.19 (1.09, 1.29)	1.32 (1.18, 1.46)	1.70 (1.56, 1.84)
GaussianCopula + Oversample	0.29 (0.25, 0.33)	1.19 (1.11, 1.26)	1.41 (1.27, 1.56)	2.40 (2.17, 2.63)
GaussanCopula Conditional	0.45 (0.31, 0.58)	0.85 (0.75, 0.96)	1.02 (0.85, 1.18)	0.51 (0.41, 0.60)
CTGAN	0.46 (0.40, 0.53)	1.37 (1.05, 1.68)	1.19 (0.96, 1.42)	1.58 (1.13, 2.03)
CTGAN Conditional	0.44 (0.37, 0.52)	0.65 (0.47, 0.82)	0.76 (0.57, 0.96)	0.53 (0.36, 0.71)
CTGAN + Oversample	0.45 (0.32, 0.57)	0.89 (0.69, 1.08)	0.93 (0.81, 1.06)	0.40 (0.28, 0.53)
TVAE	0.47 (0.42, 0.53)	0.51 (0.43, 0.58)	0.52 (0.37, 0.67)	0.70 (0.57, 0.82)
TVAE Conditional	0.52 (0.46, 0.58)	0.32 (0.23, 0.41)	0.31 (0.22, 0.40)	1.14 (1.03, 1.25)
TVAE + Oversample	0.40 (0.34, 0.45)	0.50 (0.44, 0.57)	0.53 (0.35, 0.70)	0.63 (0.53, 0.74)

Table D.18: eICU Full Northeast Results (Bootstrap)

	H_0	H_1	H_2	H_3
Locally Available Data	0.59 (0.52, 0.67)	0.38 (0.29, 0.47)	0.78 (0.74, 0.83)	0.53 (0.41, 0.65)
Combined Calibrator	0.20 (0.17, 0.23)	0.32 (0.29, 0.35)	0.79 (0.74, 0.85)	0.58 (0.46, 0.69)
Main Site Calibrator	0.59 (0.52, 0.67)	0.89 (0.81, 0.97)	1.27 (1.19, 1.34)	0.81 (0.56, 1.06)
Oversampled Data	0.61 (0.52, 0.70)	0.39 (0.30, 0.48)	0.78 (0.73, 0.83)	0.57 (0.45, 0.69)
GaussianCopula	0.53 (0.43, 0.62)	0.68 (0.46, 0.91)	0.99 (0.84, 1.15)	2.32 (1.98, 2.66)
GaussianCopula + Oversample	0.53 (0.40, 0.66)	0.50 (0.42, 0.58)	0.86 (0.68, 1.03)	2.77 (2.18, 3.35)
GaussanCopula Conditional	0.35 (0.27, 0.44)	0.62 (0.49, 0.74)	0.28 (0.23, 0.33)	1.07 (0.76, 1.38)
CTGAN	0.26 (0.20, 0.33)	0.53 (0.24, 0.82)	1.63 (1.25, 2.00)	2.70 (2.16, 3.24)
CTGAN Conditional	0.71 (0.62, 0.80)	0.51 (0.42, 0.60)	0.95 (0.84, 1.07)	0.78 (0.63, 0.93)
CTGAN + Oversample	0.29 (0.21, 0.37)	0.52 (0.37, 0.66)	0.37 (0.29, 0.45)	0.96 (0.68, 1.23)
TVAE	0.45 (0.38, 0.52)	0.55 (0.47, 0.63)	0.56 (0.50, 0.63)	0.70 (0.59, 0.81)
TVAE Conditional	0.43 (0.35, 0.51)	0.31 (0.24, 0.38)	0.70 (0.59, 0.81)	0.65 (0.45, 0.85)
TVAE + Oversample	0.42 (0.37, 0.48)	0.50 (0.42, 0.59)	0.62 (0.55, 0.69)	0.53 (0.40, 0.65)

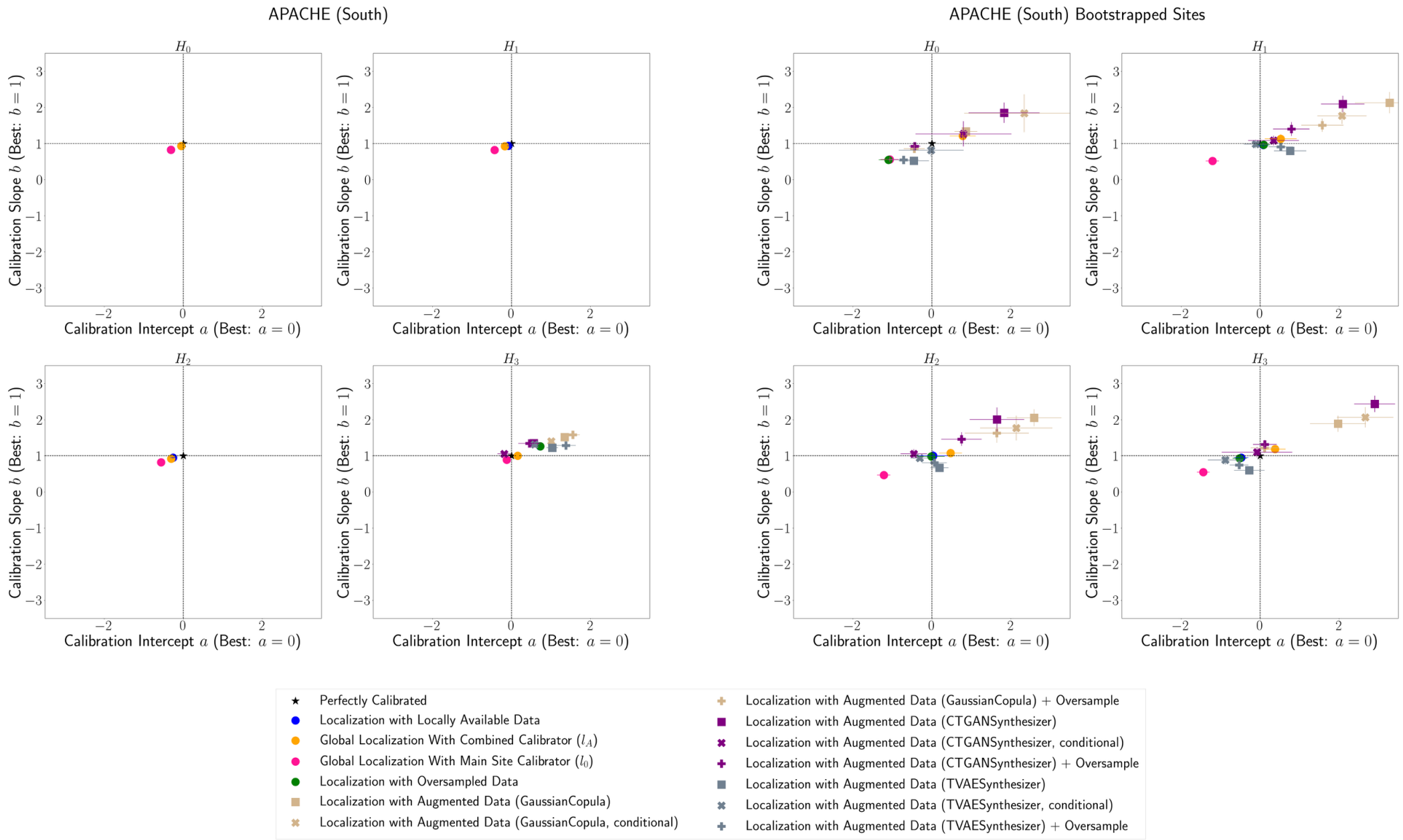

Figure D.4: Plots of slope/intercept of the calibration curve for each data augmentation method. Note how synthetic data generation approaches pushes calibration of the desired model to the ideal point (0,1), denoted by a star.

Table D.19: eICU Full South Results (Combined)

	H_0	H_1	H_2	H_3
Locally Available Data	0.36 (0.31, 0.41)	0.11 (0.08, 0.14)	0.27 (0.19, 0.34)	0.77 (0.70, 0.84)
Combined Calibrator	0.10 (0.08, 0.12)	0.19 (0.16, 0.21)	0.32 (0.26, 0.38)	0.16 (0.12, 0.21)
Main Site Calibrator	0.36 (0.31, 0.41)	0.47 (0.42, 0.51)	0.59 (0.51, 0.67)	0.20 (0.15, 0.25)
Oversampled Data	–	–	–	0.78 (0.69, 0.87)
GaussianCopula	–	–	–	1.44 (1.38, 1.51)
GaussianCopula + Oversample	–	–	–	1.09 (0.99, 1.18)
GaussanCopula Conditional	–	–	–	1.66 (1.47, 1.85)
CTGAN	–	–	–	0.72 (0.57, 0.87)
CTGAN Conditional	–	–	–	0.39 (0.27, 0.50)
CTGAN + Oversample	–	–	–	0.81 (0.63, 0.98)
TVAE	–	–	–	1.06 (0.91, 1.21)
TVAE Conditional	–	–	–	0.70 (0.58, 0.81)
TVAE + Oversample	–	–	–	1.41 (1.14, 1.68)

Table D.20: eICU Full South Results (Bootstrap)

	H_0	H_1	H_2	H_3
Locally Available Data	1.16 (0.91, 1.40)	0.32 (0.23, 0.41)	0.84 (0.62, 1.05)	0.58 (0.43, 0.73)
Combined Calibrator	0.94 (0.65, 1.24)	0.85 (0.51, 1.20)	0.72 (0.48, 0.95)	0.67 (0.47, 0.88)
Main Site Calibrator	1.16 (0.91, 1.40)	1.30 (1.11, 1.49)	1.33 (1.15, 1.51)	1.52 (1.34, 1.69)
Oversampled Data	1.19 (0.92, 1.46)	0.37 (0.26, 0.48)	0.79 (0.61, 0.98)	0.59 (0.42, 0.76)
GaussianCopula	1.12 (0.93, 1.31)	3.47 (2.50, 4.44)	2.80 (2.04, 3.57)	2.19 (1.42, 2.95)
GaussianCopula + Oversample	2.49 (0.80, 4.18)	2.21 (1.52, 2.91)	2.35 (1.35, 3.35)	2.87 (2.06, 3.68)
GaussanCopula Conditional	0.72 (0.52, 0.92)	1.68 (1.09, 2.26)	2.00 (1.19, 2.81)	0.78 (0.54, 1.02)
CTGAN	2.21 (1.29, 3.13)	2.42 (1.83, 3.01)	2.20 (1.53, 2.87)	3.24 (2.65, 3.83)
CTGAN Conditional	1.97 (0.87, 3.07)	1.15 (0.64, 1.67)	0.86 (0.65, 1.08)	1.82 (1.26, 2.39)
CTGAN + Oversample	0.49 (0.35, 0.62)	1.09 (0.64, 1.54)	1.22 (0.77, 1.67)	0.79 (0.64, 0.95)
TVAE	1.02 (0.76, 1.28)	1.15 (0.88, 1.42)	0.66 (0.55, 0.77)	1.05 (0.90, 1.19)
TVAE Conditional	1.53 (0.92, 2.14)	0.74 (0.59, 0.88)	0.62 (0.49, 0.75)	1.17 (0.82, 1.53)
TVAE + Oversample	1.05 (0.74, 1.37)	0.83 (0.60, 1.05)	0.75 (0.59, 0.91)	0.78 (0.64, 0.93)

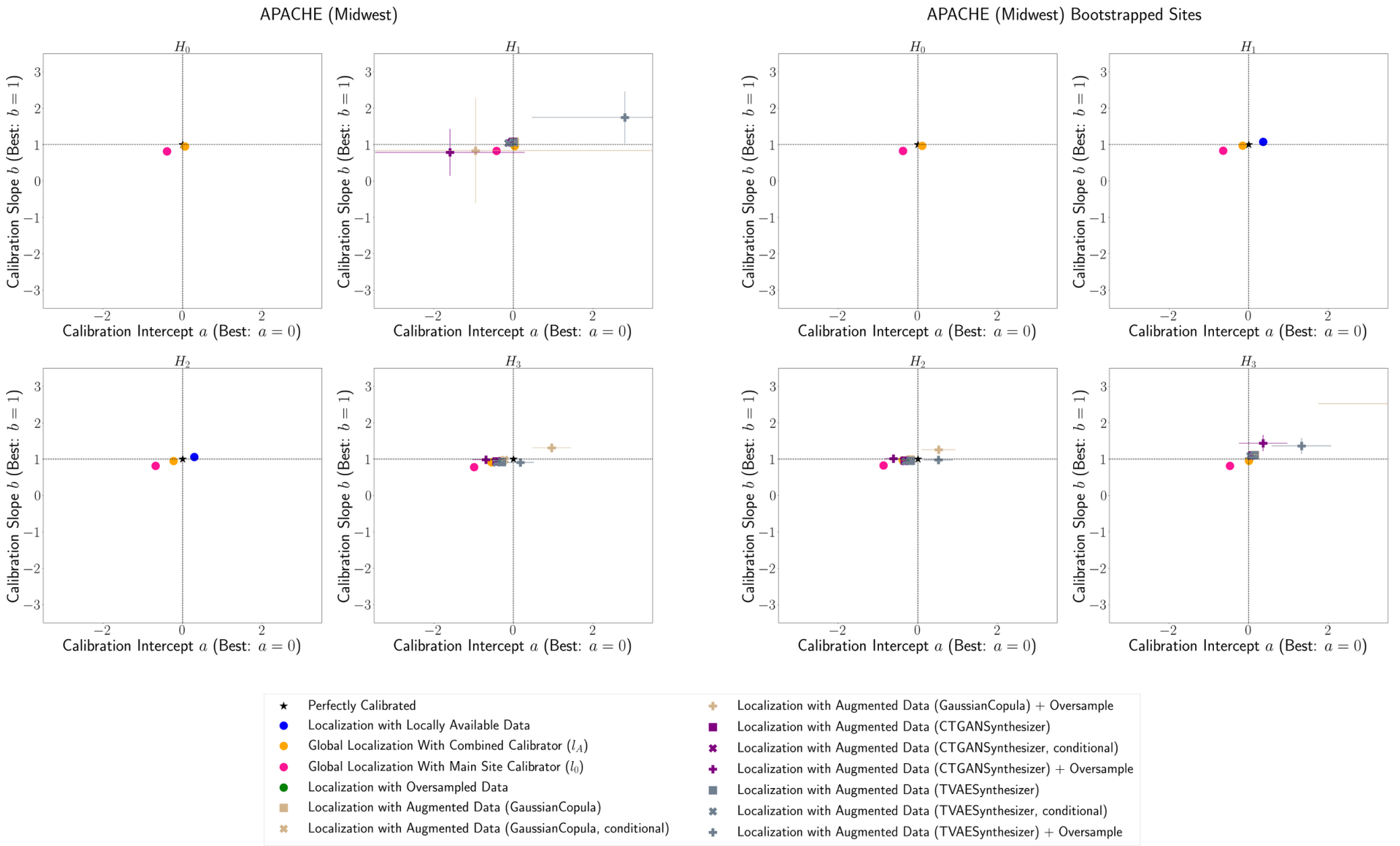

Figure D.5: Plots of slope/intercept of the calibration curve for each data augmentation method. Note how synthetic data generation approaches pushes calibration of the desired model to the ideal point (0,1), denoted by a star.

Table D.21: eICU Full Midwest Results (Combined)

	H_0	H_1	H_2	H_3
Locally Available Data	0.43 (0.38, 0.49)	0.09 (0.09, 0.10)	0.30 (0.26, 0.33)	0.36 (0.32, 0.39)
Combined Calibrator	0.08 (0.08, 0.09)	0.10 (0.08, 0.11)	0.23 (0.22, 0.24)	0.56 (0.53, 0.59)
Main Site Calibrator	0.43 (0.38, 0.49)	0.46 (0.39, 0.53)	0.70 (0.66, 0.74)	1.01 (0.95, 1.07)
Oversampled Data	–	0.09 (0.08, 0.10)	–	0.36 (0.32, 0.39)
GaussianCopula	–	0.11 (0.10, 0.12)	–	0.29 (0.25, 0.32)
GaussianCopula + Oversample	–	0.11 (0.10, 0.12)	–	0.24 (0.20, 0.28)
GaussanCopula Conditional	–	6.22 (2.03, 10.40)	–	1.23 (0.79, 1.67)
CTGAN	–	0.11 (0.10, 0.12)	–	0.43 (0.38, 0.48)
CTGAN Conditional	–	0.11 (0.09, 0.12)	–	0.45 (0.41, 0.49)
CTGAN + Oversample	–	2.46 (0.52, 4.40)	–	0.97 (0.74, 1.21)
TVAE	–	0.10 (0.09, 0.12)	–	0.30 (0.26, 0.34)
TVAE Conditional	–	0.16 (0.11, 0.22)	–	0.43 (0.38, 0.49)
TVAE + Oversample	–	3.22 (0.71, 5.73)	–	0.70 (0.47, 0.94)

Table D.22: eICU Full Midwest Results (Bootstrap)

	H_0	H_1	H_2	H_3
Locally Available Data	0.42 (0.38, 0.45)	0.37 (0.32, 0.42)	0.24 (0.22, 0.26)	0.19 (0.14, 0.25)
Combined Calibrator	0.12 (0.11, 0.14)	0.16 (0.13, 0.20)	0.38 (0.34, 0.42)	0.07 (0.06, 0.08)
Main Site Calibrator	0.42 (0.38, 0.45)	0.66 (0.61, 0.72)	0.88 (0.82, 0.94)	0.51 (0.47, 0.54)
Oversampled Data	–	–	0.26 (0.22, 0.30)	0.20 (0.15, 0.25)
GaussianCopula	–	–	0.19 (0.16, 0.22)	0.24 (0.17, 0.32)
GaussianCopula + Oversample	–	–	0.16 (0.14, 0.17)	0.21 (0.15, 0.27)
GaussanCopula Conditional	–	–	0.92 (0.57, 1.27)	4.84 (2.37, 7.32)
CTGAN	–	–	0.33 (0.28, 0.39)	0.21 (0.15, 0.27)
CTGAN Conditional	–	–	0.33 (0.30, 0.37)	0.19 (0.15, 0.24)
CTGAN + Oversample	–	–	0.74 (0.58, 0.90)	1.22 (0.70, 1.74)
TVAE	–	–	0.18 (0.14, 0.22)	0.21 (0.15, 0.27)
TVAE Conditional	–	–	0.32 (0.26, 0.38)	0.19 (0.14, 0.24)
TVAE + Oversample	–	–	0.82 (0.53, 1.11)	1.72 (1.02, 2.42)

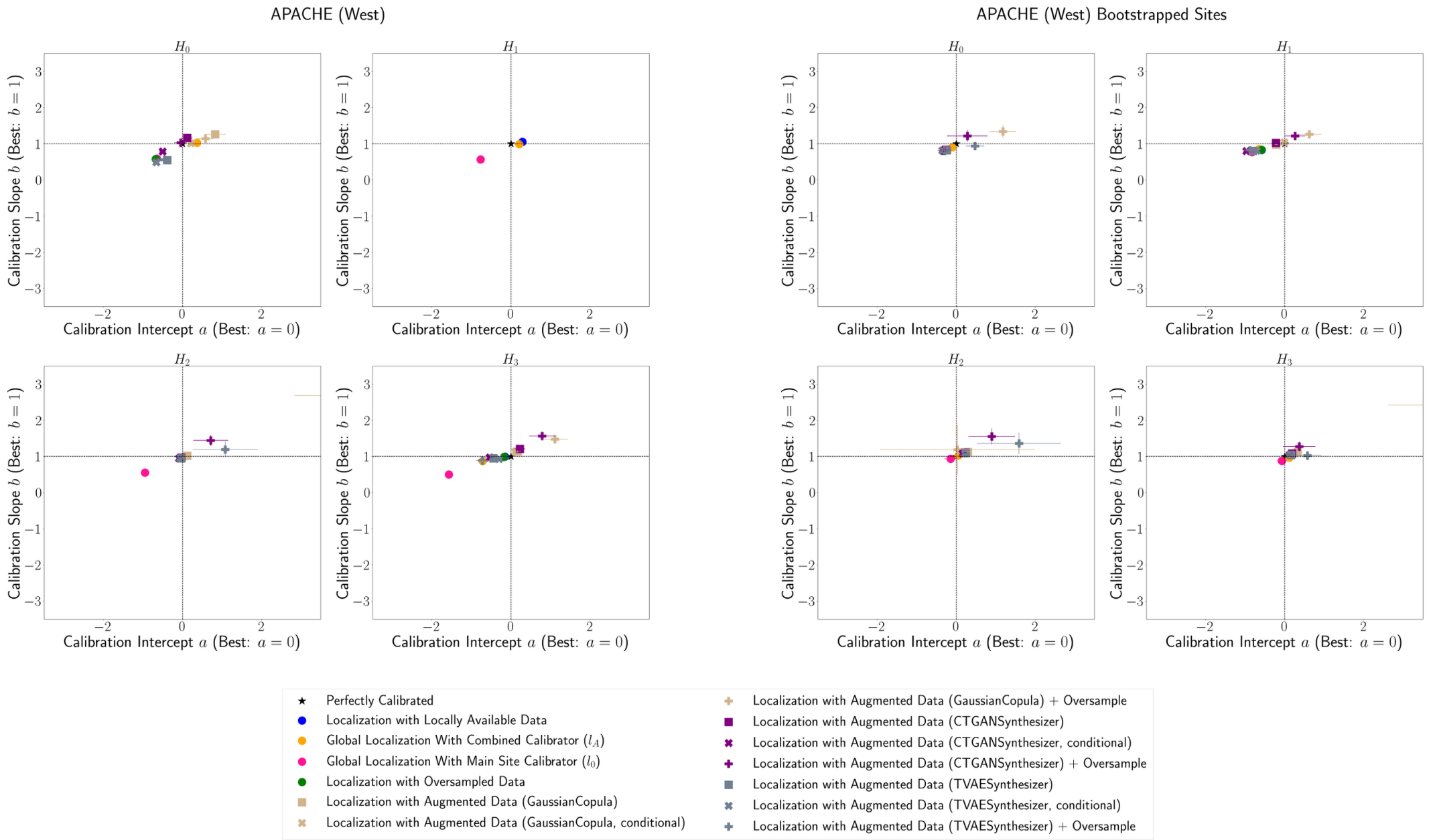

Figure D.6: Plots of slope/intercept of the calibration curve for each data augmentation method. Note how synthetic data generation approaches pushes calibration of the desired model to the ideal point (0,1), denoted by a star.

Table D.23: eICU Full West Results (Combined)

	H_0	H_1	H_2	H_3
Locally Available Data	0.77 (0.73, 0.81)	0.30 (0.25, 0.35)	0.13 (0.11, 0.16)	0.20 (0.15, 0.25)
Combined Calibrator	0.37 (0.31, 0.43)	0.22 (0.17, 0.27)	0.07 (0.06, 0.08)	0.73 (0.64, 0.82)
Main Site Calibrator	0.77 (0.73, 0.81)	0.88 (0.83, 0.93)	1.05 (1.01, 1.09)	1.65 (1.58, 1.73)
Oversampled Data	0.80 (0.76, 0.85)	–	0.14 (0.11, 0.17)	0.23 (0.17, 0.28)
GaussianCopula	0.95 (0.69, 1.20)	–	0.15 (0.10, 0.19)	0.25 (0.16, 0.34)
GaussianCopula + Oversample	0.22 (0.12, 0.32)	–	0.14 (0.11, 0.17)	0.22 (0.17, 0.27)
GaussanCopula Conditional	0.69 (0.50, 0.87)	–	5.86 (4.14, 7.58)	1.22 (0.87, 1.58)
CTGAN	0.34 (0.24, 0.45)	–	0.12 (0.09, 0.15)	0.39 (0.30, 0.49)
CTGAN Conditional	0.55 (0.44, 0.65)	–	0.14 (0.11, 0.18)	0.59 (0.50, 0.68)
CTGAN + Oversample	0.38 (0.30, 0.46)	–	1.11 (0.73, 1.50)	1.18 (0.95, 1.40)
TVAE	0.61 (0.53, 0.69)	–	0.14 (0.12, 0.17)	0.46 (0.38, 0.54)
TVAE Conditional	0.84 (0.79, 0.89)	–	0.14 (0.11, 0.16)	0.33 (0.25, 0.41)
TVAE + Oversample	0.66 (0.56, 0.75)	–	1.53 (0.75, 2.30)	0.76 (0.61, 0.91)

Table D.24: eICU Full West Results (Bootstrap)

	H_0	H_1	H_2	H_3
Locally Available Data		0.61 (0.56, 0.65)	0.23 (0.21, 0.26)	0.16 (0.14, 0.17)
Combined Calibrator	0.14 (0.12, 0.16)	0.67 (0.63, 0.71)	0.17 (0.14, 0.20)	0.13 (0.10, 0.17)
Main Site Calibrator	0.35 (0.27, 0.42)	0.86 (0.77, 0.94)	0.39 (0.28, 0.49)	0.33 (0.27, 0.40)
Oversampled Data	0.39 (0.26, 0.51)	0.60 (0.55, 0.66)	0.23 (0.20, 0.27)	0.15 (0.13, 0.17)
GaussianCopula	0.36 (0.27, 0.46)	0.22 (0.17, 0.27)	0.32 (0.29, 0.34)	0.35 (0.32, 0.38)
GaussianCopula + Oversample	0.38 (0.29, 0.48)	0.17 (0.12, 0.22)	0.32 (0.29, 0.36)	0.27 (0.25, 0.28)
GaussanCopula Conditional	1.27 (0.89, 1.65)	0.76 (0.45, 1.07)	2.60 (0.72, 4.49)	4.14 (2.74, 5.55)
CTGAN	0.38 (0.27, 0.48)	0.32 (0.23, 0.41)	0.25 (0.22, 0.29)	0.21 (0.18, 0.24)
CTGAN Conditional	0.38 (0.26, 0.50)	0.99 (0.91, 1.08)	0.17 (0.15, 0.20)	0.14 (0.12, 0.17)
CTGAN + Oversample	0.90 (0.56, 1.25)	0.65 (0.49, 0.81)	1.35 (0.79, 1.91)	0.89 (0.58, 1.21)
TVAE	0.36 (0.27, 0.46)	0.83 (0.77, 0.89)	0.26 (0.22, 0.29)	0.17 (0.15, 0.18)
TVAE Conditional	0.42 (0.29, 0.54)	0.74 (0.67, 0.81)	0.24 (0.21, 0.27)	0.17 (0.13, 0.21)
TVAE + Oversample	0.56 (0.36, 0.77)	0.93 (0.79, 1.07)	1.83 (0.73, 2.94)	0.90 (0.62, 1.17)
